# Model-informed safety management of tocilizumab for pediatric sJIA: a PBPK approach for dose-escalation and vaccination timing

**DOI:** 10.3389/fimmu.2026.1847997

**Published:** 2026-06-01

**Authors:** Yujie Yang, Rui Wang, Zhimin Li, Liang Zheng, Chaozhuang Shen

**Affiliations:** 1 Department of Pharmacy, Affiliated Hospital of Southwest Jiaotong University, The Third People’s Hospital of Chengdu, Chengdu, Sichuan, China; 2 Department of Clinical Pharmacy and Pharmacy Administration, West China school of Pharmacy, Sichuan University, Chengdu, Sichuan, China; 3 West China LeCheng Hospital of Sichuan University, Qionghai, Hainan, China; 4 Department of Pharmacy, Personalized Drug Research and Therapy Key Laboratory of Sichuan Province, Sichuan Provincial People’s Hospital, School of Medicine, University of Electronic Science and Technology of China, Chengdu, China; 5 Department of Clinical Pharmacology, The Second Affiliated Hospital of Anhui Medical University, Hefei, Anhui, China

**Keywords:** children, dose escalation, physiologically based pharmacokinetic modeling, tocilizumab, vaccines

## Abstract

**Introduction:**

Tocilizumab (TCZ), an anti-interleukin-6 receptor (IL-6R) biologic agent, is commonly used to treat systemic juvenile idiopathic arthritis (sJIA); however, the optimal dosing strategy for susceptible children and the timing of administration for safe attenuated live vaccines remain unclear. This study aimed to determine the optimal dose-escalation regimen for TCZ in children with sJIA, as well as to investigate the recommended live-vaccine schedules for pediatric patients exposed to this drug.

**Methods:**

A physiologically-based pharmacokinetic (PBPK) model integrating a target-mediated drug disposition (TMDD) structure was built for adults and extrapolated to pediatric patients.

**Results:**

The PBPK model successfully predicted and verified the pharmacokinetics (PKs) of TCZ in children aged < 2 years and 2–17 years. The predicted PK data were within two-fold of the observed data. According to the simulations, dose-escalation strategies were proposed to mitigate early hypersensitivity reactions: a 6-8-12 mg/kg sequence for patients weighing <30 kg, including infants aged <2 years and children aged 2–17 years, and a 4-6-8 mg/kg sequence for children aged 2–17 years weighing ≥30 kg. Furthermore, PBPK models indicated that, for pediatric patients of all ages, the advocated timing of live-attenuated vaccination is approximately 55 to 70 days after treatment cessation, provided that the underlying disease activity is closely monitored.

**Discussion:**

Our study illustrated that PBPK models can provide a valuable tool to predict the PKs of large macromolecules in children across the age continuum, ultimately informing precision drug-treatment decisions and vaccination regimens for the pediatric sJIA population.

## 1 Introduction

Systemic juvenile idiopathic arthritis (sJIA) is a rare, multigenic autoinflammatory disease driven by innate immunity, defined clinically by a classic triad of high fever, rash, and with or without arthritis (1, 2). The condition has been classified as a subtype of juvenile idiopathic arthritis (JIA), with a prevalence ranging from 10% to 15% among all JIA patients (3). In the early stages of sJIA, the primary immunological features manifest as inappropriate activation of the innate immune system and excessive secretion of proinflammatory cytokines such as interleukin-1, interleukin-6, and interleukin-18 (4, 5). The patients with sJIA are mainly preschool children, with an average age of onset around 6 years old, and the peak age of onset is 1 to 5 years old (6, 7). The rising incidence of pediatric sJIA poses a serious challenge to the public health system.

Immunosuppressive therapy is the key to controlling the disease activity of sJIA. The 2021 edition of the JIA ACR guidelines conditionally recommends IL-1/IL-6 inhibitors as initial monotherapy for sJIA without MAS. Data from two recent large observational studies further confirm the benefits of biologics in sJIA patients (8–10). Tocilizumab (Actemra^®^, TCZ) is a humanized IgG1 monoclonal antibody that binds both soluble and membrane-bound IL-6 receptors (sIL-6R and mIL-6R), thereby blocking IL-6–mediated signal transduction via gp130.While standard weight-based dosing regimens (12 mg/kg for<30 kg and 8 mg/kg for ≥30 kg) have demonstrated remarkable efficacy (11), the clinical application of TCZ in young pediatric populations poses multifaceted safety challenges across the treatment continuum—specifically at the initiation and cessation of therapy.

At the initiation of therapy, a primary clinical concern is the elevated risk of severe hypersensitivity and infusion-related reactions (12). This risk is particularly pronounced in infants under 2 years of age, whose immune systems are highly reactive and distinct from older pediatric cohorts (13). Although these reactions are often immune-mediated and cannot be reliably predicted by plasma concentration alone, abrupt exposure to a full initial dose may be clinically undesirable in highly vulnerable patients. Gradual dose escalation during the induction phase therefore represents a desensitization-inspired strategy to introduce TCZ more cautiously (14, 15). However, optimal dose-escalation regimens for infants lack empirical definition and are challenging to investigate in traditional clinical trials.

Conversely, treatment cessation complicates routine pediatric care, most notably the administration of live-attenuated vaccines. By blocking IL-6R signaling, TCZ diminishes primary immune responses (16, 17). To ensure safety, clinical guidelines mandate a complete drug washout before live vaccination (18) —a threshold conservatively defined in our model as systemic exposure falling below the lower limit of quantification (LLOQ), or a target occupancy low enough so as not to compromise the normal immune response to live vaccines. Predicting this exact washout period is highly complex. Driven by target-mediated drug disposition (TMDD), TCZ clearance is non-linear and heavily dependent on age-related physiology and dynamic receptor expression, rendering standard half-life estimates unreliable.

Physiologically-based pharmacokinetic (PBPK) modeling offers a robust mechanistic framework to overcome these clinical hurdles. By integrating age-dependent physiological ontogeny with disease-specific target binding kinetics, PBPK models enable reliable systemic exposure extrapolation from adults to vulnerable pediatric populations (19). Therefore, this study aimed to develop a comprehensive pediatric PBPK model of TCZ in sJIA to support model-informed dosing and vaccination-timing decisions. Specifically, we sought to (1): simulate and evaluate dose-escalation regimens for children to minimize initial hypersensitivity risks, and (2) precisely predict the individual withdrawal time required for TCZ to reach the LLOQ or a low target occupancy, thereby establishing evidence-based windows for safe immunization.

## 2 Methods

### 2.1 Software

All PBPK models were constructed using PK-Sim^®^ and MoBi^®^ (Open Systems Pharmacology Suite, version 12; www.open-systems-pharmacology.org), with plasma concentration-time profile data digitized from published literature via GetData Graph Digitizer (S. Fedorov, Version 2.25.0.32; updated to Version 2.26 for consistent data processing), and R (version 4.5, https://www.r-project.org/) software was used for data analysis and plotting.

### 2.2 Pk data

Relevant data for model development were acquired through systematic literature searches in Web of Science, PubMed, and Medline databases, using keywords such as “tocilizumab,” “pharmacokinetics,” “pharmacodynamics”, “pediatric,” “child,” “children,” “newborn,” “infant,” “adolescent,” and “teenager.” Pharmacokinetic data collected from 14 clinical studies — including four studies in healthy adults, eight studies in adult patients with rheumatoid arthritis (RA), one study in infants aged under two years, and one study in children aged two to 17 years — were segregated into a training dataset for parameter optimization and a test dataset for model evaluation. A detailed step-by-step guide to the parameter fitting procedure can be found in **Supplementary Section S1.3**. The population characteristics (race, age, and weight), dosing regimens, and baseline sIL-6R levels for each clinical study involved in model development and verification can be found in **Supplementary Table 1**.

### 2.3 Model development

#### 2.3.1 General workflow

**Figures 1:**
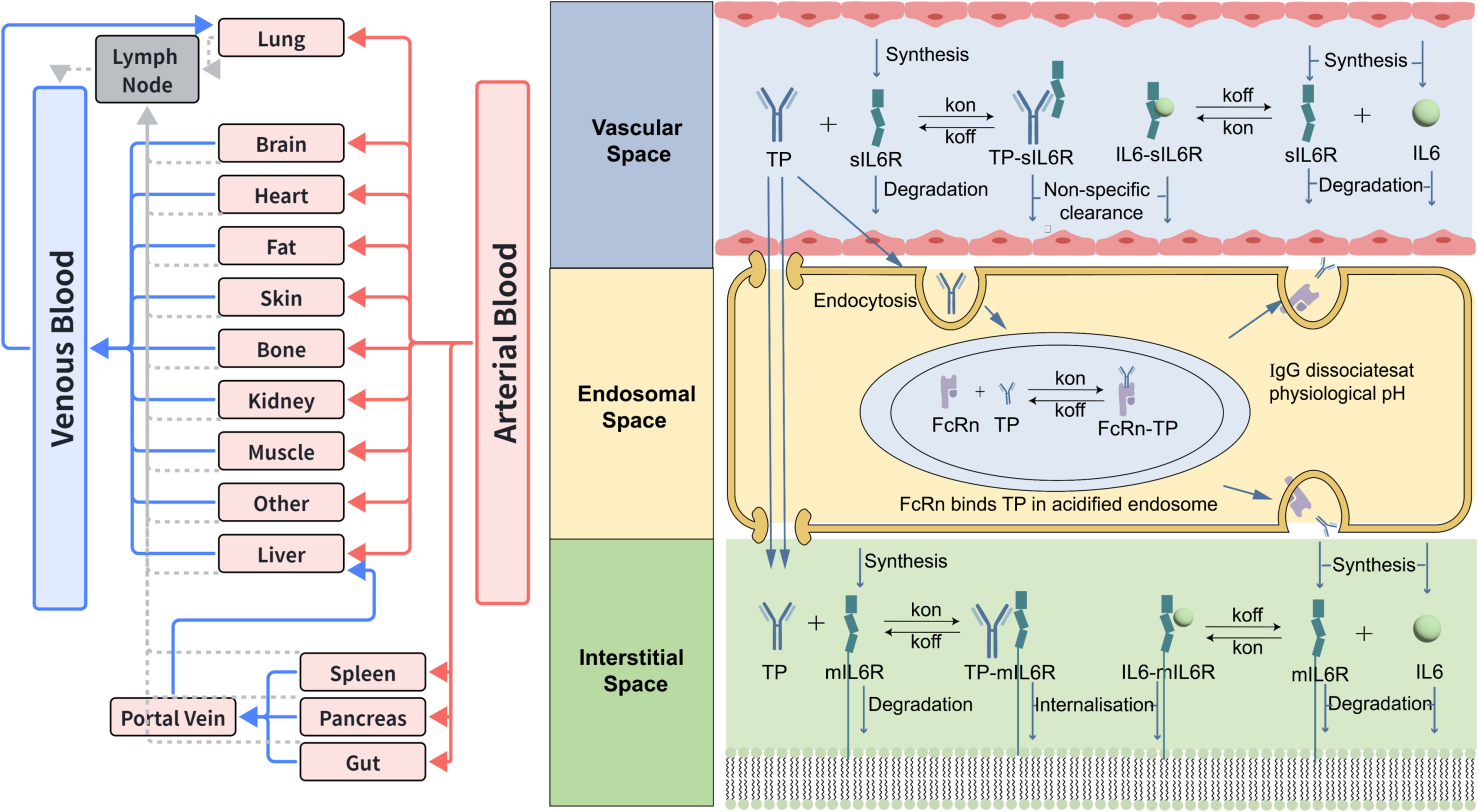
Schematic of the comprehensive PBPK modeling framework for tocilizumab. The disposition of the therapeutic protein (TP), tocilizumab, is described using a full PBPK model incorporating vascular, endosomal, and interstitial sub-spaces. In the vascular space, tocilizumab and endogenous IL-6 competitively bind to the soluble IL-6 receptor (sIL-6R). The resulting TCZ-sIL-6R complex is subsequently eliminated via general catabolic degradation following endocytosis. Following fluid-phase endocytosis into the acidic endosomal space (pH 6.0), the TP binds to the neonatal Fc receptor (FcRn), which protects it from lysosomal degradation and mediates recycling or basolateral transcytosis. Upon transport to the cell membrane and exposure to the neutral physiological environment (pH 7.4), the TP-FcRn complex immediately dissociates. In the interstitial tissue space, tocilizumab and endogenous IL-6 competitively bind to the membrane-bound receptor (mIL-6R).

This study adopted a “middle-out” strategy for developing a pediatric PBPK model of TCZ, leveraging a generic physiological framework for therapeutic proteins (**Figure 1**). The model development followed a systematic, stepwise workflow (1): establishment and verification of a base model in healthy adult volunteers (2); adaptation to adult patients with RA by incorporating disease-specific target burden; and (3) extrapolation to the pediatric sJIA population by integrating age-dependent physiological ontogeny and disease-specific cytokine baselines.

#### 2.3.2 PBPK model of Healthy and RA adults

Drug-specific parameters for TCZ were extracted from the DrugBank database (https://go.drugbank.com/) and the literature (**Table 1**). The adult PBPK model was constructed using the generic large molecule and therapeutic protein model structure available in PK-Sim (26). The virtual individual was represented by 15 organ/tissue compartments (e.g., heart, kidney, liver, lung, spleen, muscle, skin) interconnected by blood and lymph flows. Each organ compartment was further subdivided into vascular, endothelial (endosomal), interstitial, and cellular sub-compartments to mechanistically describe drug distribution. The key mechanistic processes that govern the disposition of TCZ, including FcRn-mediated recycling, the two-pore hypothesis, target-mediated drug disposition (TMDD) and disease-specific baseline parameterisations, are detailed in the **Supplementary S1** alongside the complete set of model equations.

**Table 1:** The input parameters used in PBPK modeling.

Parameter	Value	Unit	Source	Literature	Reference	Description
Tocilizumab						
MW	148,000	g/mol	Literature	148,000	Drugbnak	Molecular weight
Hydrodynamic radius	5.1	nM	PK-Sim standard	-	-	Solute radius
kup	0.31	min-1	Optimized	-	-	Rate of uptake into endosomal space
kdFcRn	460	nM	Literature	460	Chung, Shan et al.,2019 (20)	Dissociation constant for FcRn binding
KdTocilizumab-mIL-6R	0.196	nM	Literature	0.196	Xu, Christine et al.,2021 (21)	Dissociation constant for mIL6R binding
KdTocilizumab-sIL-6R	0.196	nM	Literature	0.196	Consistent with mIL6R	Dissociation constant for sIL6R binding
kintTocilizumab/mIL-6R complex	0.0186	h-1	Literature	0.0186	Xu, Christine et al.,2021 (21)	Rate constant for drug-target complex internalization
kintTocilizumab/sIL-6R complex	0.0275	h-1	Optimized	-	-	Rate constant for drug-target complex internalization
knonspecific	0.00196	h-1	Optimized	-	-	Nonspecific clearance of tocilizumab
IL6						
MW	23718	g/mol	Literature	23718	Drugbnak	Molecular weight
Hydrodynamic radius	2.2	nM	PK-Sim standard	-	-	Solute radius
kdegIL6	0.277	h-1	Literature	0.277	van Hall, Gerrit et al.,2003 (22)	Degradation rate constant for IL6
kdIL6-mIL-6R	0.08	nM	Literature	0.08	Alfinito, Eleonora et al.,2020 (23)	Dissociation constant for mIL6R binding
kdIL6-sIL-6R	0.08	nM	Literature	0.08	Consistent with mIL6R	Dissociation constant for sIL6R binding
kintIL6/mIL-6R complex	0.277	h-1	Literature	0.277	Gerhartz, C et al.,1994 (24)	Rate constant for drug-target complex internalization
kintIL6/sIL-6R complex	0.09	h-1	Optimized	-	-	Rate constant for drug-target complex internalization
Target						
kdegmIL6R	0.27	h-1	Literature	0.27	van Hall, Gerrit et al.,2003 (22)	Degradation rate constant for mIL6R
kdegsIL6R	0.36	h-1	Optimized	-	-	Degradation rate constant for sIL6R
CFcRn	a	-	Literature	a	Pan, Xian et al.,2020 (25)	FcRn concentration

mIL6R: membrane-bound IL6 receptor; sIL6R: soluble IL-6 receptor.

a:Individual FcRnpediatric (μM) = 40*(1 + 0.95*(individual IgGpediatric−mean IgGpediatric)/mean IgGpediatric)

IgGpediatric (μM) = maternal contribution + pediatric contribution

maternal contribution (μM) = 70.56 * e^−9.242 *Age(year)

pediatric contribution (μM) = 80.7*Age (year)/0.98 +Age (year)

#### 2.3.3 PBPK model of Pediatric sJIA

Given the shared pathophysiology of IL-6-driven systemic inflammation between RA and sJIA, the validated adult RA model served as the foundational framework for pediatric extrapolation (2, 11, 27). The sJIA PBPK model was derived by scaling the adult model to children using the software’s built-in ontogeny algorithms, which account for age-related changes in anatomy, blood flow, and organ maturation. In addition, the model incorporated the ontogeny of FcRn expression to accurately predict age-dependent changes in non-specific antibody clearance (25). Finally, to capture the distinct hyperinflammatory state characteristic of sJIA, the endogenous baseline levels of the target cytokines IL-6 and sIL-6R were parameterised using sJIA-specific clinical data. This clearly distinguished the pediatric sJIA population from the adult RA cohort.

#### 2.3.4 Model evaluation

The predictive performance of the PBPK model was evaluated by comparing simulated plasma concentration-time profiles with observed clinical data. Goodness-of-fit was assessed by fold-error between predicted and observed PK parameters Cmax, area under the plasma concentration-time curve (AUC0-∞, AUC0-tEnd) to evaluate the accuracy of the PBPK model according to Equation 1. The model predictions were considered acceptable if the fold error was between 0.5 and 2. The average folding error (AFE) and the absolute AFE (AAFE) of all concentration-time data points to assess the precision of the model, as shown in Equations 2, 3.

(1) $$\text{Fold error}=\frac{\text{predicted}}{\text{observed}}$$

(2) $$\text{AFE}=10^{\frac{1}{\text{n}}}\sum \log_{10}\frac{\text{predicted}}{\text{observed}}$$

(3) $$\text{AAFE}=10^{\frac{1}{\text{n}}}\sum \left|\log_{10}\frac{\text{predicted}}{\text{observed}}\right|$$

#### 2.3.5 Virtual clinical scenario simulation

Based on the established model, the timing of vaccination following the last TCZ administration was prospectively determined. Since children under 2 years of age typically weigh less than 30 kg, three virtual pediatric cohorts were created based on age and weight: children aged 0–2 years (<30kg), 2–17 years (<30kg), and 2–17 years (>30kg). Each virtual population consisted of 100 individuals. In parallel, to address safety concerns during the induction phase, our model evaluated dose-escalation regimens. Given that a minimum dose of 4 mg/kg is required to observe efficacy in pediatric patients with sJIA aged 2 to 17 years (11, 28, 29), the initial dose in all evaluation regimens was set above this threshold. Furthermore, to avoid an initial hypersensitivity reaction while preventing subtherapeutic levels, our titration protocol stipulates that if early exposure is insufficient, the dose should be increased by 2 mg/kg on top of the Week 2 dose. Based on these dosing principles, we established multiple pediatric cohorts (**Table 2**). In addition, a concentration of 0.01 μg/mL was defined as the LLOQ (30), and the time point at which the drug concentration declined to this threshold was used as an analytical drug-washout reference. To provide a more pharmacologically relevant assessment of residual IL-6R blockade, this LLOQ-based criterion was supplemented with model-predicted TCZ-mediated IL-6R receptor occupancy. Specifically, receptor occupancy below 20% was used as the primary low-residual-blockade benchmark, and a more conservative threshold of below 10% was further evaluated to reduce uncertainty in the model-informed assessment of live-attenuated vaccination timing.

**Table 2:** Simulated AUC_0–2weeks_ and Week 1 Cmax of proposed tocilizumab dose-escalation regimens in children with sJIA.

Strategy Study	AUC_0-2week_[umol*min/l]			Cmax^a^ [umol/l]		
	Child < 2year	Child 2-17 year, Weight < 30Kg	Child 2-17 year, Weight > 30Kg	Child < 2year	Child 2-17 year, Weight < 30Kg	Child 2-17 year, Weight > 30Kg
Strategy 1	8318.85	9088.01	-	74.01	74.54	-
Strategy 2	8608.33	9302.89	-	92.52	93.18	-
Strategy 3	8897.21	9649.52	-	111.03	111.82	-
Strategy 4	9548.08	10419.16	-	74.01	74.54	-
Strategy 5	9838.46	10634.59	-	92.52	93.18	-
Strategy 6	10128.21	11115.86	-	111.03	111.82	-
Strategy 7	-	-	7708.79	-	-	90.22
Strategy 8	-	-	9291.71	-	-	90.22
Model predicted	10821.41	11730.73	9409.42	221.39	222.28	180.49

AUC_0-2week_, Area under the concentration vs. time curve from the first to 2 weeks.

^a^Cmax refers to the maximum predicted serum tocilizumab concentration during Week 1 following the initial dose.

“Strategies” refer to the simulated dose-escalation regimens intended to mitigate early hypersensitivity. Doses are sequentially administered at Week 1 (initial dose), Week 2 (second dose), and Week 3 onwards (maintenance dose).

Strategy 1: 4 mg/kg, 8 mg/kg, and 12 mg/kg.

Strategy 2: 5 mg/kg, 7 mg/kg, and 12 mg/kg.

Strategy 3: 6 mg/kg, 6 mg/kg, and 12 mg/kg.

Strategy 4: 4 mg/kg, 10 mg/kg, and 12 mg/kg.

Strategy 5: 5 mg/kg, 9 mg/kg, and 12 mg/kg.

Strategy 6: 6 mg/kg, 8 mg/kg, and 12 mg/kg.

Strategy 7: 4 mg/kg, 4 mg/kg, and 8 mg/kg.

Strategy 8: 4 mg/kg, 6 mg/kg, and 8 mg/kg.

Model predicted: conventional non-escalated dosing every 2 weeks, with doses administered at Week 1, Week 3, Week 5, and so forth. The dose was 12 mg/kg for patients weighing<30 kg, including infants aged<2 years, and 8 mg/kg for patients weighing ≥30 kg.

## 3 Results

### 3.1 Development and validation of the PBPK model for healthy adults and RA

**Figure 2:**
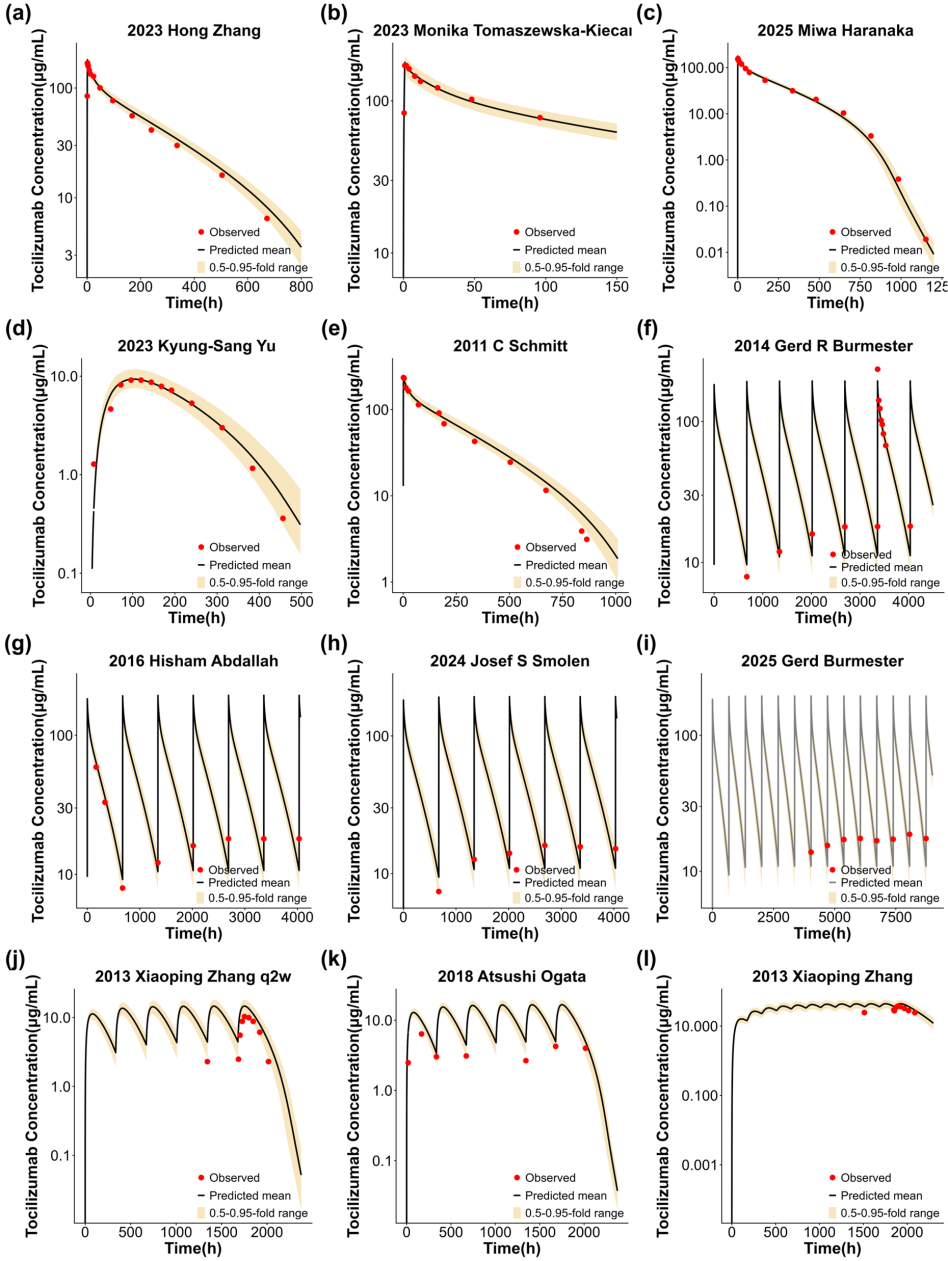
PBPK model-predicted versus observed concentration-time profiles of tocilizumab in healthy adults and rheumatoid arthritis (RA) patients. Panels **(a–d)** represent healthy adults receiving a single intravenous (IV) infusion of 8 mg/kg **(a–c)** or a single subcutaneous (SC) injection of 162 mg **(d)**. Panels **(e–l)** represent RA patients receiving a single IV infusion of 10 mg/kg **(e)**, multiple IV infusions of 8 mg/kg every 4 weeks (q4w) **(f–i)**, multiple SC injections of 162 mg every 2 weeks (q2w) **(j, k)**, or multiple SC injections of 162 mg every week (qw) **(l)**. The solid gray lines indicate the predicted mean of the PBPK model, the yellow shaded areas represent the 90% prediction intervals, and the red dots represent the observed data.

**Figure 3:**
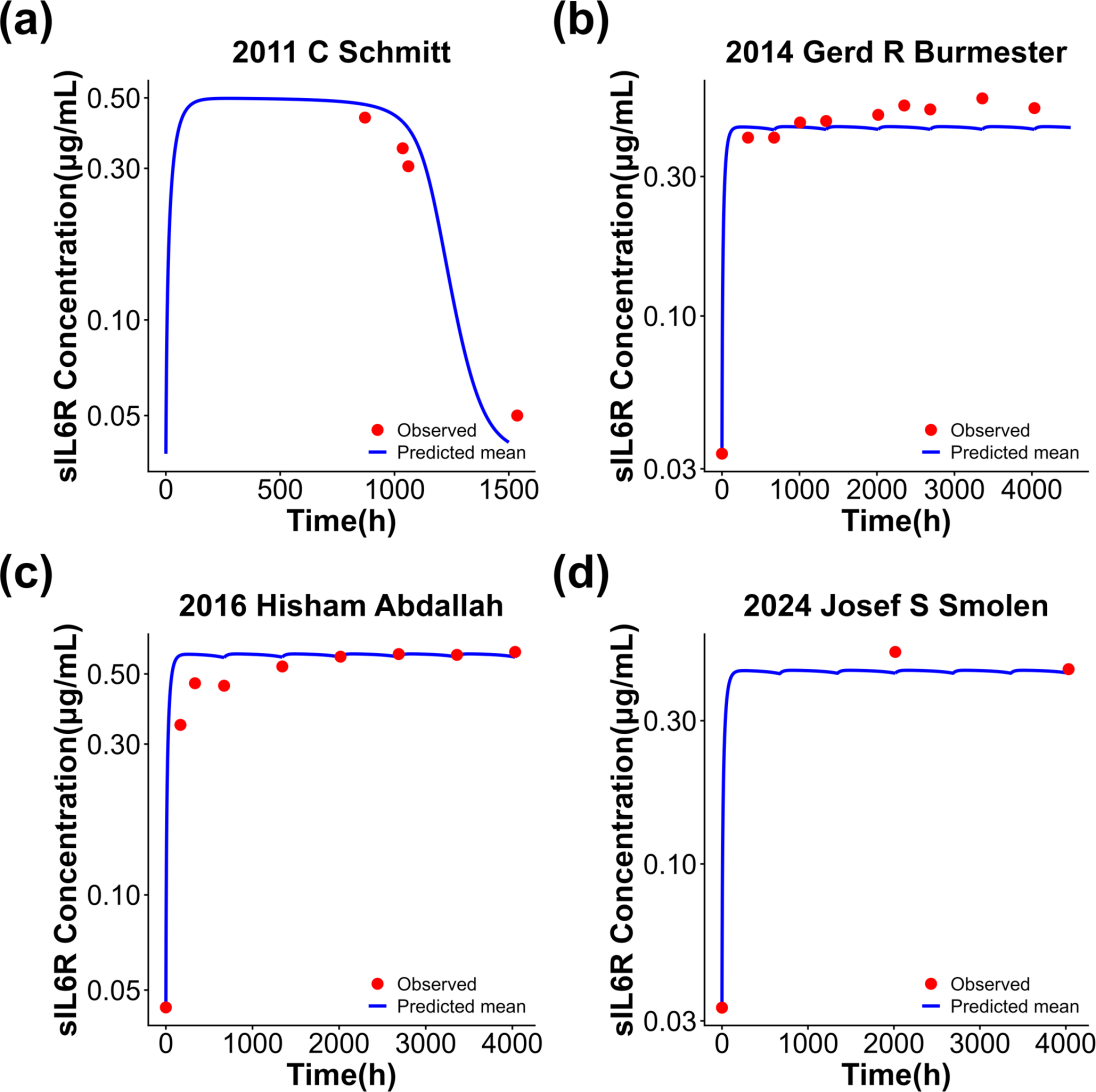
PBPK model-predicted versus observed concentration-time profiles of total sIL-6R following tocilizumab administration. The panels represent different dosing regimens and populations: **(a)** a single intravenous infusion of 10 mg/kg; **(b–d)** 8 mg/kg every 4 weeks (q4w). The solid lines represent the predicted mean of the PBPK model, and the symbols represent observed data.

**Figure 4:**
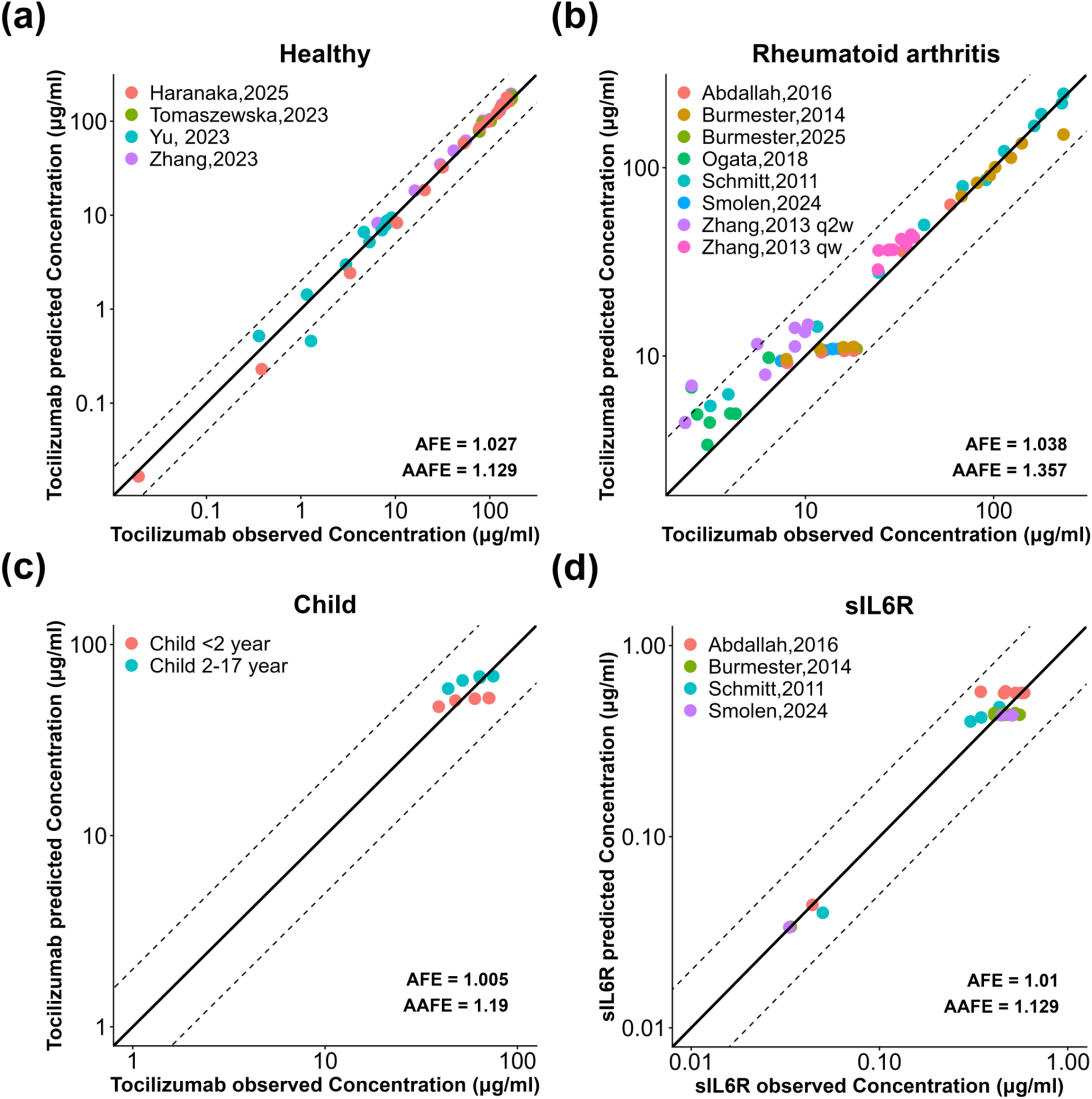
PBPK model-predicted versus observed concentration-time points of tocilizumab in healthy adults **(a)**, rheumatoid arthritis (RA) patients **(b)**, and pediatric systemic juvenile idiopathic arthritis (sJIA) patients **(c)**, as well as total sIL-6R **(d)**. The solid black line indicates the unity line, and the dashed black lines indicate the two-fold error margins.

The developed base PBPK model accurately described the concentration-time profiles of tocilizumab (TCZ) in healthy adult volunteers and RA patients (**Figure 2**). By integrating TMDD mechanisms, the model successfully captured the non-linear clearance kinetics driven by sIL-6R and mIL-6R. Correspondingly, the model accurately simulated the temporal dynamics and characteristic accumulation of total sIL-6R concentrations (**Figure 3**). Furthermore, the incorporation of disease-specific baseline levels of IL-6 and sIL-6R enabled the model to demonstrate robust goodness-of-fit across various dosing regimens (intravenous and subcutaneous administration) in the RA cohort. Quantitative evaluations confirmed that the predicted-to-observed ratios (fold errors) for primary pharmacokinetic parameters (Cmax,AUC0-∞) strictly fell within the predefined two-fold acceptance criterion (0.5 to 2.0) (**Supplementary Table 2**; **Figure 4**).

### 3.2 Extrapolation and evaluation of the pediatric sJIA model

**Figure 5:**
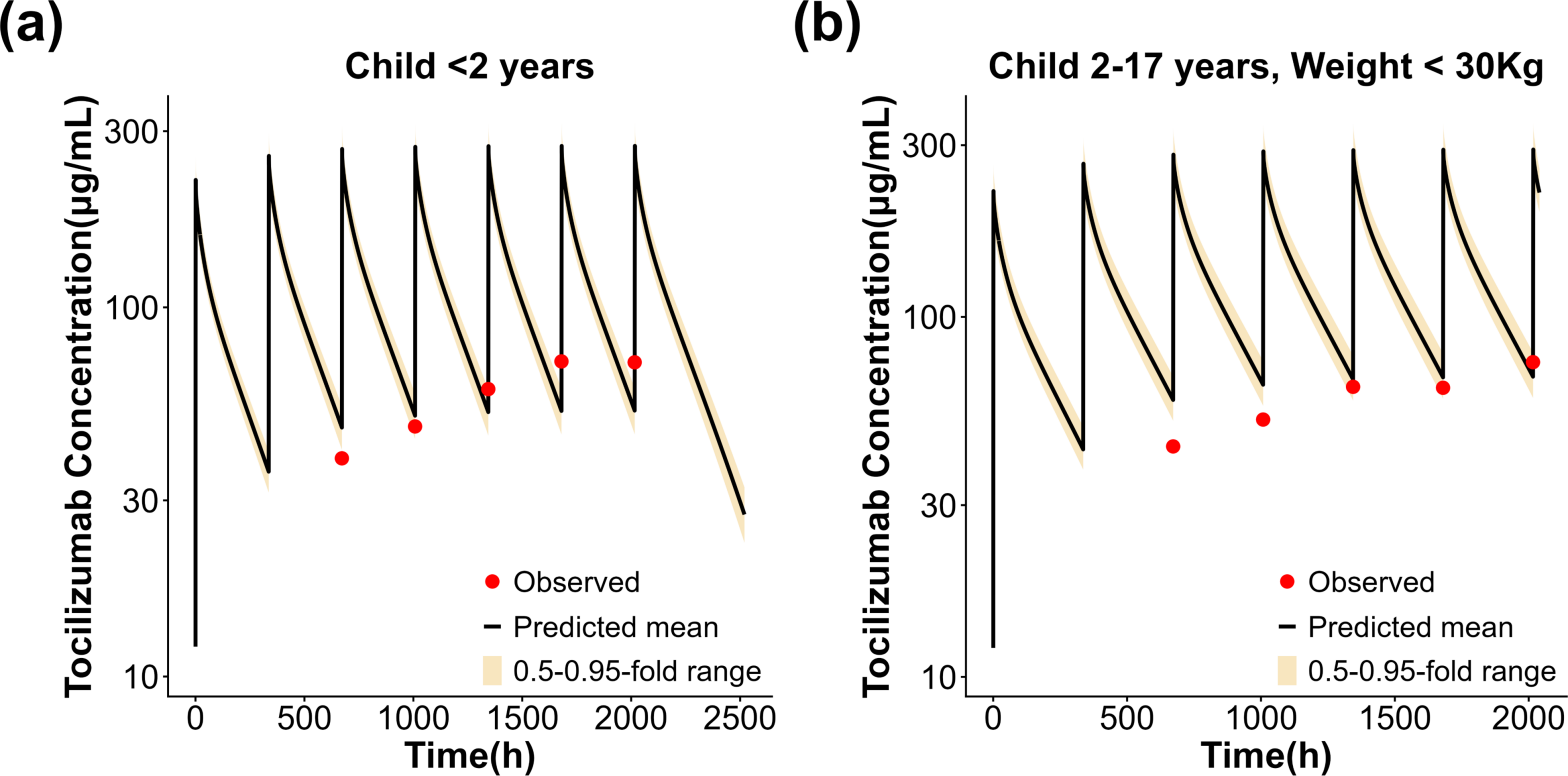
Population simulation of tocilizumab (TCZ) following intravenous administration of 12 mg/kg in pediatric sJIA patients. Panels represent patients aged< 2 years weighing< 30 kg **(a)**, and patients aged 2–17 years weighing< 30 kg **(b)**. The solid gray line indicates the predicted mean of the PBPK model; the yellow shaded area represents the 90% model prediction interval; red dots, observed values of tocilizumab.

This study successfully constructed a pediatric sJIA extrapolation model based on the validated adult RA model without additional parameter optimization. This model integrates ontogeny algorithms (particularly those precisely describing the age-dependent maturation of FcRn expression) with sJIA-specific cytokine baselines. The model accurately predicted TCZ exposure in pediatric sJIA patients in clinical settings (**Figure 5**). Visual Prediction Check (VPC) confirmed that the vast majority of clinical observation points were uniformly distributed within the 90% prediction interval (i.e., the 5th to 95th percentiles). Further quantitative analysis of model accuracy revealed AFE and AAFE values of 1.005 and 1.19, respectively, indicating extremely low systematic bias and high predictive precision within the target pediatric population (**Figure 4**). However, it should be noted that the available clinical validation data were limited to pediatric patients weighing<30 kg. Therefore, predictions for the ≥30 kg cohort were not directly validated and should be interpreted as model-based extrapolations.

### 3.3 Simulation and optimization of dose-escalation regimens across pediatric cohorts

To address the elevated risk of immune-mediated hypersensitivity in vulnerable pediatric cohorts, the model simulated the pharmacokinetic profiles of various dose-escalation induction regimens. As demonstrated, all evaluated dose-escalation strategies successfully reduced the Week 1 Cmax compared with the conventional standard-dose regimen, indicating a clear attenuation of the abrupt initial peak exposure. However, initial evaluations revealed that the preliminary step-up strategies (Strategies 1–3, 7) resulted in suboptimal cumulative early exposure (AUC) across all age and weight groups. Consequently, a targeted adjustment was applied by specifically elevating the second dose (Week 2) to compensate for the rapid target-mediated clearance.

Following this second-dose elevation, the AUC performance improved significantly across all evaluated strategies (**Table 2**). Notably, for both infants< 2 years and children aged 2–17 years weighing< 30 kg, the dose-escalation strategy consisting of 6 mg/kg for the initial dose, 8 mg/kg at Week 2, and 12 mg/kg for subsequent maintenance emerged as the optimal regimen, demonstrating the best AUC performance. A parallel necessity to escalate the second dose was also confirmed for the ≥ 30 kg cohort, where an optimized sequence of 4 mg/kg for the initial dose, 6 mg/kg at Week 2, and 8 mg/kg for maintenance was established to secure adequate early exposure.

### 3.4 Prediction of safe vaccination windows post-cessation

**Figure 6:**
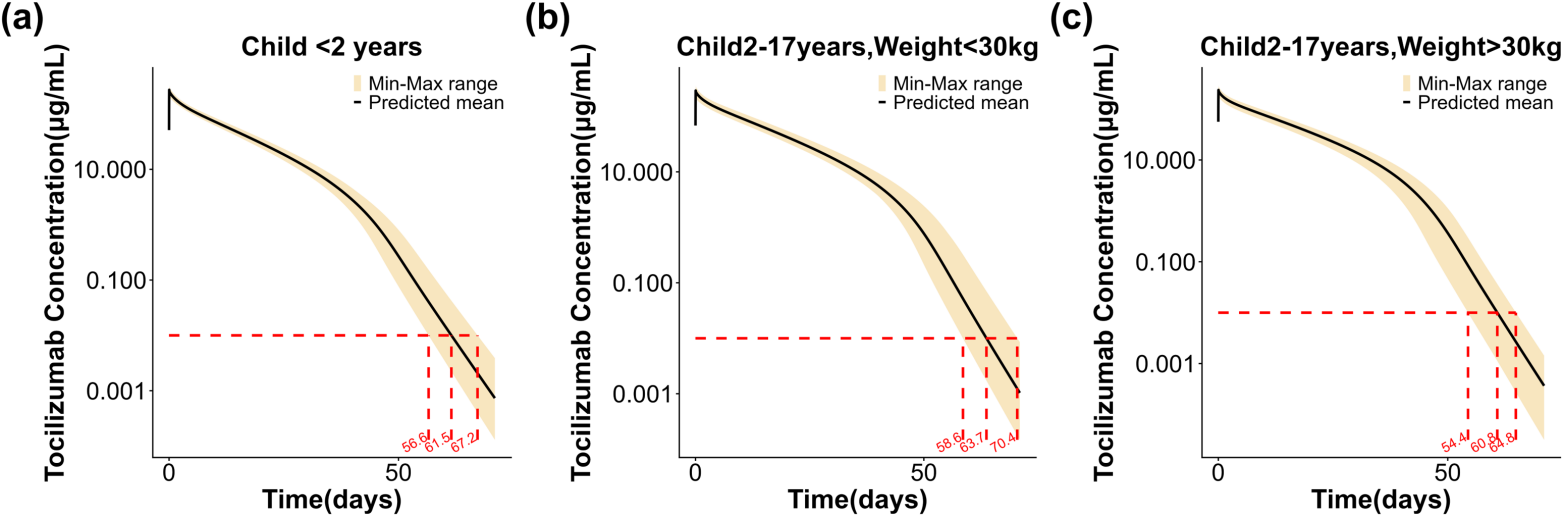
Simulated pharmacokinetic profiles for predicting live-attenuated vaccination timing and evaluating dose-escalation strategies in pediatric sJIA patients. Panels **(a–c)** illustrate the concentration-time profiles used to determine the optimal washout period for live-attenuated vaccines following the cessation of optimized tocilizumab dose-escalation therapy in patients aged< 2 years (6-8–12 mg/kg regimen, a), aged 2–17 years weighing< 30 kg (6-8–12 mg/kg regimen, **(b)**, and aged 2–17 years weighing ≥ 30 kg (4-6–8 mg/kg regimen, **(c)**. The solid gray line indicates the predicted mean of the PBPK model; the yellow shaded area represents the Min-Max model prediction interval.

To guide the optimal timing for administering attenuated live vaccines to pediatric patients with sJIA, this study utilized a validated pediatric PBPK model to simulate the washout period following discontinuation of steady-state TCZ dosing. Following the discontinuation of therapy, simulation results demonstrated a high degree of consistency across all three pediatric cohorts, with the mean time for serum TCZ concentrations to fall below the LLOQ at approximately 55 to 70 days (**Figure 6**). Specifically, for infants< 2 years (weight< 30 kg) treated with the optimized 6-8–12 mg/kg dose-escalation regimen, the mean washout time was 61.5 days (Min–Max: 56.6–67.2 days); for children aged 2–17 years (weight< 30 kg) receiving the same optimized regimen, it was 63.7 days (Min–Max: 58.6–70.4 days); and for children aged 2–17 years (weight ≥ 30 kg) treated with the optimized 4-6–8 mg/kg regimen, it was 60.8 days (Min–Max: 54.4–64.8 days). To further contextualize residual IL-6R blockade at the time of pharmacological washout, TCZ-mediated IL-6R receptor occupancy was also evaluated. Across all pediatric cohorts, the estimated time for receptor occupancy to decline below 20% was approximately 1 day longer than the LLOQ-based washout time, whereas the time to decline below 10% was approximately 4 days longer (**Supplementary Figure 3**).

In addition, we simulated the washout period following discontinuation of treatment for patients who had already begun receiving the standard treatment regimen (**Supplementary Figure 1**). Notably, consistent with the optimized regimen, the predicted elimination times across the three pediatric cohorts remained highly consistent; it still took approximately 55 to 70 days to safely reduce drug levels below the lower dose threshold. The receptor occupancy-based analysis showed the same temporal pattern, with the 20% occupancy criterion extending the predicted washout time by approximately 1 day and the 10% occupancy criterion by approximately 4 days relative to the LLOQ-based estimate (**Supplementary Figure 4**).

## 4 Discussion

To our knowledge, this study provides the first comprehensive, mechanism-based physiologically based pharmacokinetic (PBPK) modeling framework for tocilizumab (TCZ) specifically developed to cover the entire pediatric age continuum in systemic juvenile idiopathic arthritis (sJIA). By mechanistically integrating TMDD kinetics, FcRn-mediated recycling ontogeny, and disease-specific inflammatory baselines, our model successfully extrapolated systemic exposure from adults to children. Furthermore, the model addressed two critical unmet clinical needs in pediatric rheumatology: optimizing dose-escalation regimens for infants to mitigate hypersensitivity, and quantifying the precise washout period to inform safe vaccination scheduling.

Although Pan et al. (31) successfully predicted metabolic therapeutic protein-drug interactions (TP-DIs) in adult RA patients using dynamic receptor binding kinetics, this study provides a clearer description of the TMDD process by explicitly distinguishing between mIL-6R and sIL-6R receptors. In model simulations, we observed that the total sIL-6R concentration gradually increased and plateaued following TCZ administration, subsequently returning to baseline levels as TCZ was eliminated from the body—a pattern consistent with clinical observations. This likely occurs because TCZ binds to free sIL-6R post-administration, forming a drug-target complex. The elimination rate of this complex (kintTocilizumab/sIL-6R complex) is significantly lower than that of free sIL-6R (kdegsIL6R), leading to a markedly prolonged residence time of the complex in the systemic circulation. Concurrently, the body continuously generates new sIL-6R, resulting in a corresponding increase in total sIL-6R concentration. When the generation rate of free sIL-6R reaches a dynamic equilibrium with the elimination rate of the TCZ-sIL-6R complex, the concentration increase slows and enters a plateau phase. During the washout phase, as TCZ is gradually eliminated from the body, newly generated sIL-6R is no longer bound, and existing complexes gradually dissociate and clear. Consequently, total sIL-6R concentration ultimately returns to normal baseline levels (32, 33).

Standard full-dose initiation may result in abrupt early systemic TCZ exposure in highly vulnerable pediatric patients (12); however, hypersensitivity and infusion-related reactions to monoclonal antibodies are often immune-mediated and cannot be reliably inferred from plasma exposure alone (34). Therefore, the dose-escalation strategies evaluated in our study—specifically the 6-8–12 mg/kg sequence (Weeks 1, 2, and maintenance) for patients< 30 kg, including infants< 2 years, and the 4-6–8 mg/kg sequence for patients ≥ 30 kg—should be interpreted as desensitization-inspired induction regimens designed to reduce the abruptness of early exposure while progressively approaching the approved maintenance dose. By increasing the second dose at Week 2, these regimens attenuated the simulated initial concentration peak and maintained early cumulative exposure (AUC_0–2 weeks_) within the reference exposure range predicted for standard TCZ regimens. Nevertheless, because the initial fractionated dose is lower than the conventional starting dose, inflammation control during the first few days may be transiently reduced. Accordingly, close clinical monitoring of at-risk pediatric patients for signs of incomplete disease control is recommended during the first week before administration of the compensatory second dose.

Previous empirical studies have yielded conflicting evidence regarding vaccine responses during TCZ therapy. Although TCZ may reduce humoral immune responses, other studies indicate that TCZ does not significantly impair antibody responses to T-cell-dependent or T-cell-independent antigens, such as trivalent inactivated influenza vaccine (TIV) (35), 23-valent pneumococcal polysaccharide vaccine (PPV23) (36), or tetanus toxoid vaccine (TTV) (37). However, these studies primarily evaluated inactivated vaccines administered only after short-term (e.g., two infusions) TCZ exposure, leaving the protective immunity threshold during long-term treatment undefined.

More importantly, live-attenuated vaccines, such as MMR and varicella vaccines, should be avoided during active biologic immunosuppression, as stated in the Actemra^®^ prescribing information (38). The EULAR/PRES recommendations similarly emphasize caution with live-attenuated vaccines in pediatric patients receiving biologic DMARDs (39). Current clinical guidelines offer varying timelines for post-treatment vaccination. For instance, the 2021 ACR JIA guidelines, citing CDC recommendations, broadly advise deferring live vaccines for 1 to 6 months after the cessation of immunosuppressive therapy (40). Meanwhile, other consensus statements recommend a withholding period of up to 2 months post-treatment (18). To address this variability, our model-based simulations provide the quantitative precision necessary to mechanistically refine these empirical recommendations. Due to the concentration-dependent shortening of the half-life—driven by the underlying TMDD mechanism—TCZ is eliminated more rapidly as systemic concentrations decline. Our results demonstrate a highly consistent mean washout period of approximately 60 days (~2 months) to fall below the LLOQ-based analytical washout reference. When supplemented by receptor-occupancy analysis, the time to low residual IL-6R blockade was only modestly longer, with receptor occupancy declining below 20% approximately 1 day later and below 10% approximately 4 days later. Thus, the model-derived 9- to 10-week withholding period after the final TCZ dose is broadly consistent with the shorter end of existing 1- to 6-month guidance and with recommendations suggesting avoidance of live vaccines for up to 2 months after treatment cessation. However, this estimate should be interpreted as a model-informed pharmacological washout window rather than a validated clinical safety threshold for live-attenuated vaccination, and therefore requires future clinical validation.

This study had several limitations. First, pharmacokinetic data for pediatric populations remain scarce overall, with particularly limited empirical data available for newborns and infants under two years of age. Furthermore, existing clinical validation data for ages 2–17 spans an overly broad age range, lacking representative data for specific age cohorts. In addition, the available clinical validation data primarily supported the<30 kg cohorts, whereas the ≥30 kg cohort was not directly validated; therefore, the proposed 4-6–8 mg/kg regimen for patients weighing ≥30 kg should be interpreted as an extrapolative model-based recommendation requiring further confirmation. Second, the current model relies on fixed, disease-specific baseline parameterizations for sIL-6R. Physiologically, sIL-6R is primarily generated via the proteolytic shedding of mIL-6R mediated by the ADAM17 enzyme, whose activity is dramatically upregulated during severe hyperinflammatory states (41). Consequently, our static baseline approach may not fully capture the transient, massive sIL-6R target burden that occurs during acute disease exacerbations. Finally, due to the absence of a definitively reported dissociation constant for TCZ binding specifically to sIL-6R, our model assumed this affinity to be equivalent to that for mIL-6R (21). While structural homology supports this assumption, any subtle deviations in the true binding kinetics could marginally influence the precise prediction of the TCZ-sIL-6R complex elimination rate, underscoring the need for integrating dynamic disease-state mechanisms and richer pediatric sampling in future research.

## 5 Conclusion

This study developed a pediatric PBPK model that successfully simulated the plasma concentration distribution of tocilizumab in sJIA patients under 2 years old and those aged 2–17 years.It explored model-informed dose-escalation regimens for vulnerable pediatric patients and proposed a 9 to 10 week post-discontinuation washout period for safe live-vaccine administration. These findings provide insights for establishing safe and effective clinical dosing and vaccination strategies in pediatric sJIA populations.

## Data Availability

The original contributions presented in the study are included in the article/**Supplementary Material**. Further inquiries can be directed to the corresponding author.
