# Supplementary material for: Model-informed safety management of tocilizumab for pediatric sJIA: a PBPK approach for dose-escalation and vaccination timing
Source: Front Immunol. 2026 Jun 1;17:1847997. doi: 10.3389/fimmu.2026.1847997 (PMC13265457; doi:10.3389/fimmu.2026.1847997)
Supplement: Supplementary file 1 [file Table1.docx]

***Supplementary Material***

# **Methods (Addendum)**

## Virtual individuals

The PBPK model was developed primarily based on clinical data from adults and children and was constructed using the mean values for gender, age, weight, height, and body mass index (BMI) reported in each study protocol. For adult individuals where specific demographic information was not reported, default characteristics from the PK-Sim® population database were substituted: male, European, 30 years of age, 73 kg body weight, 176 cm body height, and 23.57 kg/m² BMI. For pediatric cohorts, virtual individuals were constructed using the actual mean values of age and weight reported in each clinical study protocol. In cases where the ethnicity of the pediatric population was not explicitly stated, the European population was assumed.

As tocilizumab is a monoclonal antibody targeting the soluble interleukin-6 receptor (sIL-6R), the baseline concentration of the target is a critical driver of target-mediated drug disposition (TMDD). When baseline sIL-6R levels were not reported in the studies, a typical endogenous sIL-6R level of 50 ng/mL (equivalent to 0.97 nM, based on a sIL-6R molecular mass of 51.5 kDa)(1) was utilized as the physiological reference.

## Virtual populations

For population development and the simulation of dose-escalation strategies, virtual populations containing 100 individuals were created for each cohort. These populations were based on the characteristics described in the respective study protocols. System-dependent parameters, including organ weights, blood flow, and tissue composition—all of which vary with age, sex, weight, and BMI—were assigned by the population generation algorithm to incorporate natural biological variability(2).

## Parameter Optimization Strategies

To ensure the identifiability of the target-mediated drug disposition (TMDD) model, parameter optimization was conducted using a highly controlled, biologically-driven approach. The clinical data were divided into a training dataset for parameter estimation and a validation dataset for extrapolation assessment (the specific allocation for each study is detailed in Supplementary Table 1).

To avoid the non-identifiability issues typically associated with the simultaneous optimization of multiple rate constants, a stepwise fitting procedure was employed. Given that tocilizumab specifically binds to the D3 extracellular domain—which is structurally identical in both the soluble IL-6 receptor (sIL-6R) and the membrane-bound IL-6 receptor (mIL-6R)—the well-documented kinetic parameters of mIL-6R were utilized as physiological references. Initially, target-specific parameters governing the disposition of sIL-6R and its interaction with the drug (e.g., complex internalization kint and receptor degradation kdeg) were optimized. The parameter bounds for these values were strictly constrained to remain within approximately one order of magnitude of the literature-reported mIL-6R data. Following the stabilization of the target-related backbone, the non-specific clearance (knonspecific) and systemic uptake (kup) were subsequently refined.

The parameter estimation was executed using the Monte Carlo algorithm within the modeling software. The optimization process was configured with the following specific parameters: a maximum number of iterations of 10000; a scale of projection degree set to 30; and a break condition for relative error improvement established at 0.001.

## Key Mechanisms and Structural Assumptions of the TCZ PBPK Model

To accurately simulate the biodistribution and complex nonlinear elimination of tocilizumab (TCZ), we structurally modified the universal macromolecular framework within the Open Systems Pharmacology (OSP) component of MoBi®. Key mechanistic processes and physiological assumptions governing TCZ disposition include:

(1)Convection and Diffusion: Describing the movement of the large molecule between plasma and interstitial space via the two-pore hypothesis(3,4).

(2)Transcytosis and Recycling: Determining the uptake into the endosomal space and the protection from catabolic degradation via binding to the neonatal Fc receptor (FcRn).

(3)Target-Mediated Drug Disposition (TMDD): To mechanistically capture the non-linear pharmacokinetics of TCZ, a full competitive TMDD model was implemented via custom structural modifications in MoBi®. This component explicitly incorporates the dynamic binding competition between TCZ and endogenous IL-6 for both sIL-6R and mIL-6R. This component describes the association, dissociation, and internalization/degradation kinetics of TCZ-mIL-6R and TCZ-sIL-6R complexes. It assumes that IL6 and sIL6R generation and elimination occur only in plasma.

(4)Healthy and RA: For the transition from healthy adults to patients with RA, physiological parameters were assumed to remain unchanged, with the exception of the baseline concentrations of IL-6 and sIL-6R. Preliminary sensitivity analyses indicated that variations in baseline IL-6 levels exert minimal influence on the overall pharmacokinetics of TCZ. Therefore, we fixed the endogenous baseline IL-6 concentration in healthy subjects at 5 pg/mL. For RA patients, disease-specific IL-6 baseline parameters utilized values reported in relevant clinical trials (or defaulted to a representative 50 pg/mL when empirical data were unavailable). Similarly, the baseline systemic sIL-6R concentration in healthy adults was set at 50 ng/mL(1),while the rheumatoid arthritis patient cohort was assigned study-specific baseline sIL-6R levels.

(5)In the absence of directly comparable isoform-specific affinity data, the model assumed that IL-6 and TCZ bind sIL-6R and mIL-6R with the same affinity. This assumption was based on the fact that sIL-6R and mIL-6R share the extracellular IL-6Rα domains responsible for ligand and antibody binding(5,6). IL-6 primarily interacts with the D2-D3 cytokine-binding module of IL-6Rα(5), and recent structural data indicate that TCZ binds the D3 domain of IL-6Rα(6). By contrast, the main structural differences between sIL-6R and mIL-6R are located in the membrane-proximal stalk, transmembrane, and intracellular regions(7), which are not expected to directly alter the core IL-6/TCZ binding interface. Therefore, the base model used Kd_IL6−sIL6R_ =Kd,_IL6−mIL6R_ and Kd,_TCZ−sIL6R_=Kd,_TCZ−mIL6R_. Given the potential importance of this assumption for TCZ–sIL-6R complex formation, sIL-6R accumulation, and terminal TCZ elimination, the robustness of model predictions to this assumption was assessed through formal sensitivity analyses.

## Mathematical Equations for Therapeutic Protein and Cytokine Receptor Kinetics

This section provides the mathematical framework for modelling cytokine receptor antagonist therapeutic protein (TP), cytokine, and (membrane-bound and soluble) cytokine receptor kinetics.

① Free drug concentration in peripheral venous blood as shown in Equation (1)

$\frac{\text{d}\text{C}_{\text{TP}}}{\text{dt}}\text{=-}\text{k}_{\text{non}}\text{⋅}\text{C}_{\text{TP}}\text{-}\text{k}_{\text{on,TP,s}}\text{⋅}\text{C}_{\text{TP}}\text{⋅}\text{C}_{\text{sR}}\text{+}\text{k}_{\text{off,TP,s}}\text{⋅}\text{R}\text{C}_{\text{s,TP}}\text{-}\text{k}_{\text{on,TP,m}}\text{⋅}\text{C}_{\text{TP}}\text{⋅}\text{C}_{\text{mR}}\text{+}\text{k}_{\text{off,TP,m}}\text{⋅}\text{R}\text{C}_{\text{m,TP}}$

(1)

② Free IL-6 concentration in peripheral venous blood as shown in Equation (2)

$\frac{\text{d}\text{C}_{\text{IL6}}}{\text{dt}}\text{=}\text{k}_{\text{syn,IL6}}\text{-}\text{k}_{\text{deg,IL6}}\text{⋅}\text{C}_{\text{IL6}}\text{-}\text{k}_{\text{on,IL6,s}}\text{⋅}\text{C}_{\text{IL6}}\text{⋅}\text{C}_{\text{sR}}\text{+}\text{k}_{\text{off,IL6,s}}\text{⋅}\text{R}\text{C}_{\text{s,IL6}}\text{-}\text{k}_{\text{on,IL6,m}}\text{⋅}\text{C}_{\text{IL6}}\text{⋅}\text{C}_{\text{mR}}\text{+}\text{k}_{\text{off,IL6,m}}\text{⋅}\text{R}\text{C}_{\text{m,IL6}}$ (2)

③ Free soluble IL-6 receptor concentration in peripheral venous blood as shown in Equation (3)

$\frac{\text{d}\text{C}_{\text{sR}}}{\text{dt}}\text{=}\text{k}_{\text{syn,sR}}\text{-}\text{k}_{\text{deg,sR}}\text{⋅}\text{C}_{\text{sR}}\text{-}\text{k}_{\text{on,TP,s}}\text{⋅}\text{C}_{\text{TP}}\text{⋅}\text{C}_{\text{sR}}\text{+}\text{k}_{\text{off,TP,s}}\text{⋅}\text{R}\text{C}_{\text{s,TP}}\text{-}\text{k}_{\text{on,IL6,s}}\text{⋅}\text{C}_{\text{IL6}}\text{⋅}\text{C}_{\text{sR}}\text{+}\text{k}_{\text{off,IL6,s}}\text{⋅}\text{R}\text{C}_{\text{s,IL6}}$ (3)

④ Total membrane-bound IL-6 receptor concentration in tissue interstitial space is defined in equation (4)

$\frac{\text{d}\text{C}_{\text{mR}}}{\text{dt}}\text{=}\text{k}_{\text{syn,mR}}\text{-}\text{k}_{\text{deg,mR}}\text{⋅}\text{C}_{\text{mR}}\text{-}\text{k}_{\text{on,TP,m}}\text{⋅}\text{C}_{\text{TP}}\text{⋅}\text{C}_{\text{mR}}\text{+}\text{k}_{\text{off,TP,m}}\text{⋅}\text{R}\text{C}_{\text{m,TP}}\text{-}\text{k}_{\text{on,IL6,m}}\text{⋅}\text{C}_{\text{IL6}}\text{⋅}\text{C}_{\text{mR}}\text{+}\text{k}_{\text{off,IL6,m}}\text{⋅}\text{R}\text{C}_{\text{m,IL6}}$ (4)

⑤ Free tocilizumab-soluble IL-6 receptor complex concentration in peripheral venous blood as shown in Equation (5)

$\frac{\text{dR}\text{C}_{\text{s,TP}}}{\text{dt}}\text{=}\text{k}_{\text{on,TP,s}}\text{⋅}\text{C}_{\text{TP}}\text{⋅}\text{C}_{\text{sR}}\text{-}\text{k}_{\text{off,TP,s}}\text{⋅}\text{R}\text{C}_{\text{s,TP}}\text{-}\text{k}_{\text{int,TP,s}}\text{⋅}\text{R}\text{C}_{\text{s,TP}}\text{+}\text{k}_{\text{on,IL6,s}}\text{⋅}\text{C}_{\text{IL6}}\text{⋅}\text{C}_{\text{sR}}\text{-}\text{k}_{\text{off,IL6,s}}\text{⋅}\text{R}\text{C}_{\text{s,IL6}}\text{-}\text{k}_{\text{int,IL6,s}}\text{⋅}\text{R}\text{C}_{\text{s,TP}}$ (5)

⑥ Tocilizumab-membrane-bound IL-6 receptor complex in tissue interstitial space is defined in Equation (6)

$\frac{\text{dR}\text{C}_{\text{m,TP}}}{\text{dt}}\text{=}\text{k}_{\text{on,TP,m}}\text{⋅}\text{C}_{\text{TP}}\text{⋅}\text{C}_{\text{mR}}\text{-}\text{k}_{\text{off,TP,m}}\text{⋅}\text{R}\text{C}_{\text{m,TP}}\text{-}\text{k}_{\text{int,TP,m}}\text{⋅}\text{R}\text{C}_{\text{m,TP}}\text{+}\text{k}_{\text{on,IL6,m}}\text{⋅}\text{C}_{\text{IL6}}\text{⋅}\text{C}_{\text{sR}}\text{-}\text{k}_{\text{off,IL6,m}}\text{⋅}\text{R}\text{C}_{\text{s,IL6}}\text{-}\text{k}_{\text{int,IL6,m}}\text{⋅}\text{R}\text{C}_{\text{s,TP}}$ (6)

⑦ Tocilizumab-mediated IL-6 receptor occupancy is defined in Equation (7)

$$RO_{\text{s}\text{IL6R}}\left( t \right)=\frac{\text{R}\text{C}_{\text{s,TP}}}{\text{R}\text{C}_{\text{s,TP}}+C_{sR}}\times100\%$$

Variable Definitions:

· $\text{C}_{\text{TP}}$: Free tocilizumab concentration

· $\text{C}_{\text{IL6}}$: Free endogenous IL-6 concentration

· $\text{C}_{\text{sR}}$: Free soluble IL-6 receptor concentration

· $\text{C}_{\text{mR}}$: Free membrane-bound IL-6 receptor concentration

· $\text{R}\text{C}_{\text{s,TP}}$: Concentration of the tocilizumab-soluble IL-6 receptor complex

· $\text{R}\text{C}_{\text{m,TP}}$: Concentration of the tocilizumab-membrane-bound IL-6 receptor complex

· $\text{R}\text{C}_{\text{s,IL6}}$: Concentration of the IL-6 and soluble IL-6 receptor complex

· $\text{R}\text{C}_{\text{m,IL6}}$: Concentration of the IL-6 and membrane-bound IL-6 receptor complex

· $\text{k}_{\text{on,TP,s}}$: Rate constant for tocilizumab binding to soluble IL-6 receptor

· $\text{k}_{\text{off,TP,s}}$: Rate constant for dissociation of tocilizumab-soluble IL-6 receptor complex

· $\text{k}_{\text{on,TP,m}}$: Rate constant for tocilizumab binding to membrane-bound IL-6 receptor

· $\text{k}_{\text{off,TP,m}}$: Rate constant for dissociation of tocilizumab-membrane-bound IL-6 receptor complex

· $\text{k}_{\text{non}}$: Rate constant for nonspecific clearance of tocilizumab

· $\text{k}_{\text{on,IL6,s}}$: Rate constant for IL-6 binding to soluble IL-6 receptor

· $\text{k}_{\text{off,IL6,s}}$: Rate constant for dissociation of IL-6 and soluble IL-6 receptor complex

· $\text{k}_{\text{on,IL6,m}}$: Rate constant for IL-6 binding to membrane-bound IL-6

· $\text{k}_{\text{off,IL6,m}}$: Rate constant for dissociation of IL-6 and membrane-bound IL-6 receptor complex

· $\text{k}_{\text{syn,IL6}}$: IL-6 synthesis rate

· $\text{k}_{\text{deg,IL6}}$: Degradation rate constant for IL-6

· $\text{k}_{\text{syn,sR}}$: Soluble IL-6 receptor synthesis rate

· $\text{k}_{\text{deg,sR}}$: degradation rate constant for soluble IL-6 receptor

· $\text{k}_{\text{syn,mR}}$: Membrane-bound IL-6 receptor synthesis rate

· $\text{k}_{\text{deg,mR}}$: Degradation rate constant for membrane-bound IL-6 receptor

· $\text{k}_{\text{int,TP,}\text{s}}$: Rate constant for tocilizumab-soluble IL-6 receptor complex internalisation

· $\text{k}_{\text{int,TP,m}}$: Rate constant for tocilizumab-membrane-bound IL-6 receptor complex internalisation

· $\text{k}_{\text{int,IL6,s}}$: Rate constant for IL-6 and soluble IL-6 receptor complex internalisation

· $\text{k}_{\text{int,IL6,m}}$: Rate constant for IL-6 and membrane-bound IL-6 receptor complex internalisation

# **Supplementary Figures and Tables**

## Supplementary Table 1 Data sets used for evaluation of PK predictions

| **Study** | **Dose** | **Population** | **N** | **Female[%]** | **Age, years** | **Weight, kg** | **Hight, cm** | **Dataset** | **sIL6R** |
| --- | --- | --- | --- | --- | --- | --- | --- | --- | --- |
| **Healthy** | | | | | | | |  |  |
| Yu, Kyung-Sang et al., 2023(8) | SC single-dose 162mg | European adult | 143 | 24.5 | 26 [19–51] | 69.5 [51.3–98.0] | 172.1 [153.3–197.7] | training | NA |
| Haranaka, Miwa et al.,2025(9) | IV single-dose 8mg/kg | Japanese adult | 44 | 0 | 26.50 [18-54] | 61.55 [51.80-85.60] | 169.40 [161.70-182.00] | training | NA |
| Tomaszewska-Kiecana, Monika et al.,2023(10) | IV single-dose 8mg/kg | European adult | 66 | 28.8 | 32.8 ± 9 | 76.53 ± 10.1 | NA | test | NA |
| Zhang, Hong et al.,2021(11) | IV single-dose 8mg/kg | Chinese adult | 42 | NA | 36.1 ± 8.51 | 66.21 ± 7.621 | NA | test | NA |
| **Rheumatoid arthritis** | | | | | | | |  |  |
| Schmitt, C et al.,2011(12) | IV single-dose 10mg/kg | American adult | 12 | 66.7 | 56 [28–72] | 88 [63-111] | NA | test | 37.8788 |
| Burmester, Gerd et al.,2025(13) | IV multiple 4-weekly 8mg/kg doses for 28 weeks | European adult | 109 | 67.9 | 57.0 [27–73] | 75.85 ± 18.037 | NA | training | 33.6 ± 13.02 |
| Smolen, Josef S et al.,2024(14) | IV multiple 4-weekly 8mg/kg doses for 24 weeks | European adult | 237 | 75.9 | 55.0 [22–73] | NA | NA | training | 33.6 ± 13.02 |
| Abdallah, Hisham et al.,2017(15) | IV multiple 4-weekly 8mg/kg doses for 24 weeks | Adults from multiple countries | 631 | 82.6 | 52.8 ± 12.53 | 74.4 ± 19.01 | NA | test | 44 |
| Burmester, Gerd R et al.,2014(16) | IV multiple 4-weekly 8mg/kg doses for 24 weeks | Adults from multiple countries | 537 | 82.7 | 52.5 ± 12.50 | 73.82 ± 18.99 | NA | test | 33.8078 |
| Ogata, Atsushi et al.,2018(17) | SC multiple 2-weekly 162 mg doses for 12 weeks | Japanese adult | 20 | 70 | 55.1 ± 12.8 | 65.9 ± 14.4 | NA | training | NA |
| Zhang, Xiaoping et al.,2013(18) | SC multiple 2-weekly 162 mg doses for 12 weeks | American adult | 15 | 87 | 54.7 ± 12.4 | 80.1 ± 13.2 | NA | training | 50.47 |
| Zhang, Xiaoping et al.,2013(18) | SC multiple 1-weekly 162 mg doses for 12 weeks | American adult | 14 | 50 | 58.2 ± 10.8 | 82.0 ± 15.9 | NA | test | NA |
| **Systemic juvenile idiopathic arthritis** | | | | | | | |  |  |
| Mallalieu, Navita L et al.,2019(19) | IV multiple 2-weekly 12mg/kg doses for 24 weeks | Adults from multiple countries | 11 | 63.6 | 1.3 [0.83-1.83] | 9.97 ± 1.38 | NA | test | 39.4322 |
| Mallalieu, Navita L et al.,2019(19) | IV multiple 2-weekly 12mg/kg doses for 24 weeks | Adults from multiple countries | 38 | 47 | 6.6 [2-16] | 20.07 ± 5.93 | NA | test | 39.4322 |

Data presented as mean or median and [range], (interquartile range), or ± SD;

NA, not applicable, N, sample size

## Supplementary Table 2 Comparison of PBPK model-predicted and observed PK parameters of tocilizumab

| Study |  | AUC_0-∞_ [µmol*min/l] | AUC_0-tEnd_ [µmol*min/l] | C_max [µmol/l] |
| --- | --- | --- | --- | --- |
| Healthy | | | | |
| Yu, Kyung-Sang et al., 2023 | Observed | 858.72 | 849.74 | 0.0616 |
|  | Predicted | 900.26 | 892.41 | 0.0631 |
|  | Fold error | 1.05 | 1.05 | 1.02 |
| Haranaka, Miwa et al.,2025 | Observed | 11750.13 | 11749.70 | 1.20 |
|  | Predicted | 11680.8 | 11680.55 | 1.06 |
|  | Fold error | 0.99 | 0.99 | 1.13 |
| Tomaszewska-Kiecana, Monika et al.,2023 | Observed | 9635.61 | 4167.26 | 1.15 |
|  | Predicted | 12575.45 | 5595.09 | 1.21 |
|  | Fold error | 1.31 | 1.34 | 1.05 |
| Zhang, Hong et al.,2023 | Observed | 11739.8 | 11253.26 | 1.14 |
|  | Predicted | 12484.77 | 12301.72 | 1.22 |
|  | Fold error | 1.06 | 1.09 | 1.07 |
| Rheumatoid arthritis | | | | |
| Schmitt, C et al.,2011 | Observed | 16711.35 | 16573.54 | 1.59 |
|  | Predicted | 18069.86 | 18069.76 | 1.67 |
|  | Fold error | 1.08 | 1.09 | 1.05 |
| Systemic juvenile idiopathic arthritis | | | | |
| Mallalieu, Navita L et al.,2019^a^ | Observed | - | 8729.75^c^ | 1.95 |
|  | Predicted | - | 10821.41^d^ | 1.82 |
|  | Fold error | - | 0.81 | 0.93 |
| Mallalieu, Navita L et al.,2019^b^ | Observed | - | 12214.61^c^ | 1.77 |
|  | Predicted | - | 11730.73^d^ | 1.94 |
|  | Fold error | - | 0.96 | 1.1 |

a: Child<2year; b:Child 2-17 year; c: Observed data area under the concentration–time curve to 2 weeks; d: Predicted: data area under the concentration–time curve to 2 weeks

AUC_0-∞_ : Area under the concentration Vs. time curve from the first data point extrapolated to infinity.

AUC_0-tEnd_ : Area under the concentration vs. time curve from the first to the last data point

## Supplementary Figure 1


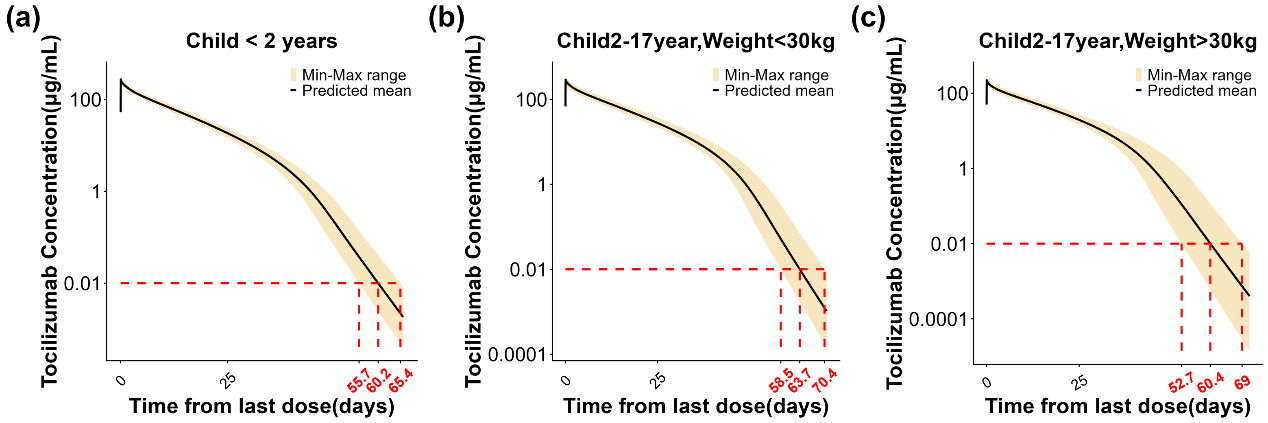


**Supplementary Figure 1.** Simulated pharmacokinetic profiles for predicting live-attenuated vaccination timing following the cessation of conventional standard dosing in pediatric sJIA patients. Panels (a–c) illustrate the concentration-time profiles used to determine the optimal washout period for live-attenuated vaccines following the early cessation of conventional standard tocilizumab therapy in patients aged < 2 years (12 mg/kg standard regimen, a), aged 2–17 years weighing < 30 kg (12 mg/kg standard regimen, b), and aged 2–17 years weighing ≥ 30 kg (8 mg/kg standard regimen, c). The solid grey line indicates the predicted mean of the PBPK model; the yellow shaded area represents the Min-Max model prediction interval.

## Supplementary Figure 2


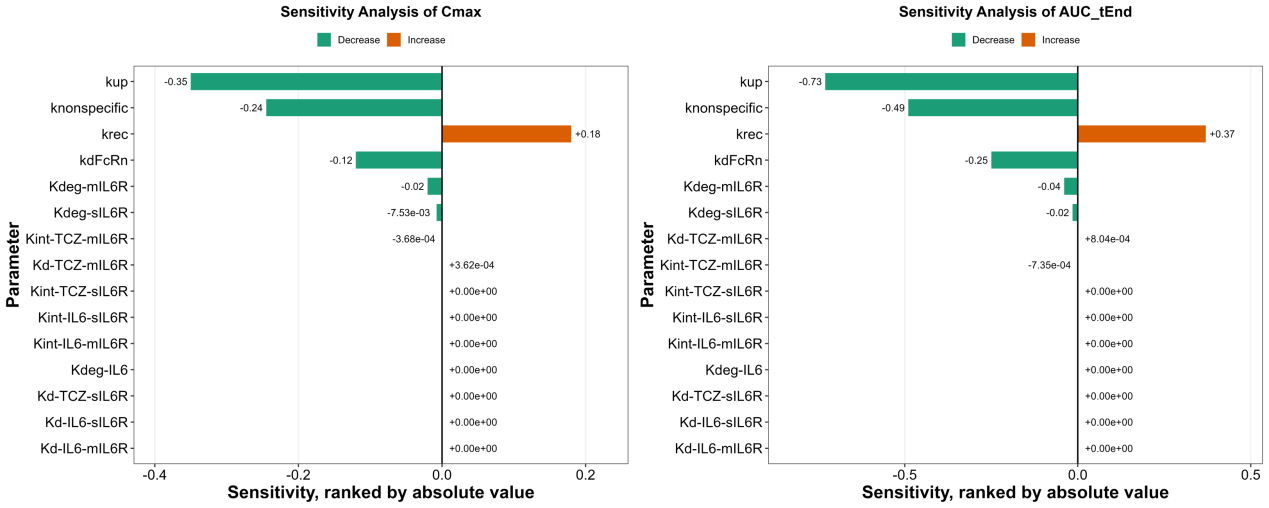


**Supplementary Figure 2.**Sensitivity analysis of TMDD parameters on Cmax and AUC_tEnd. A sensitivity value of +1.0 signifies that a 10% increase of the examined parameter causes a 10% increase of the simulated AUC0-t and Cmax. Cmax represents the maximum plasma concentration, and AUC_tEnd represents the area under the plasma concentration–time curve from the first to the last simulated data point.

## Supplementary Figure 3


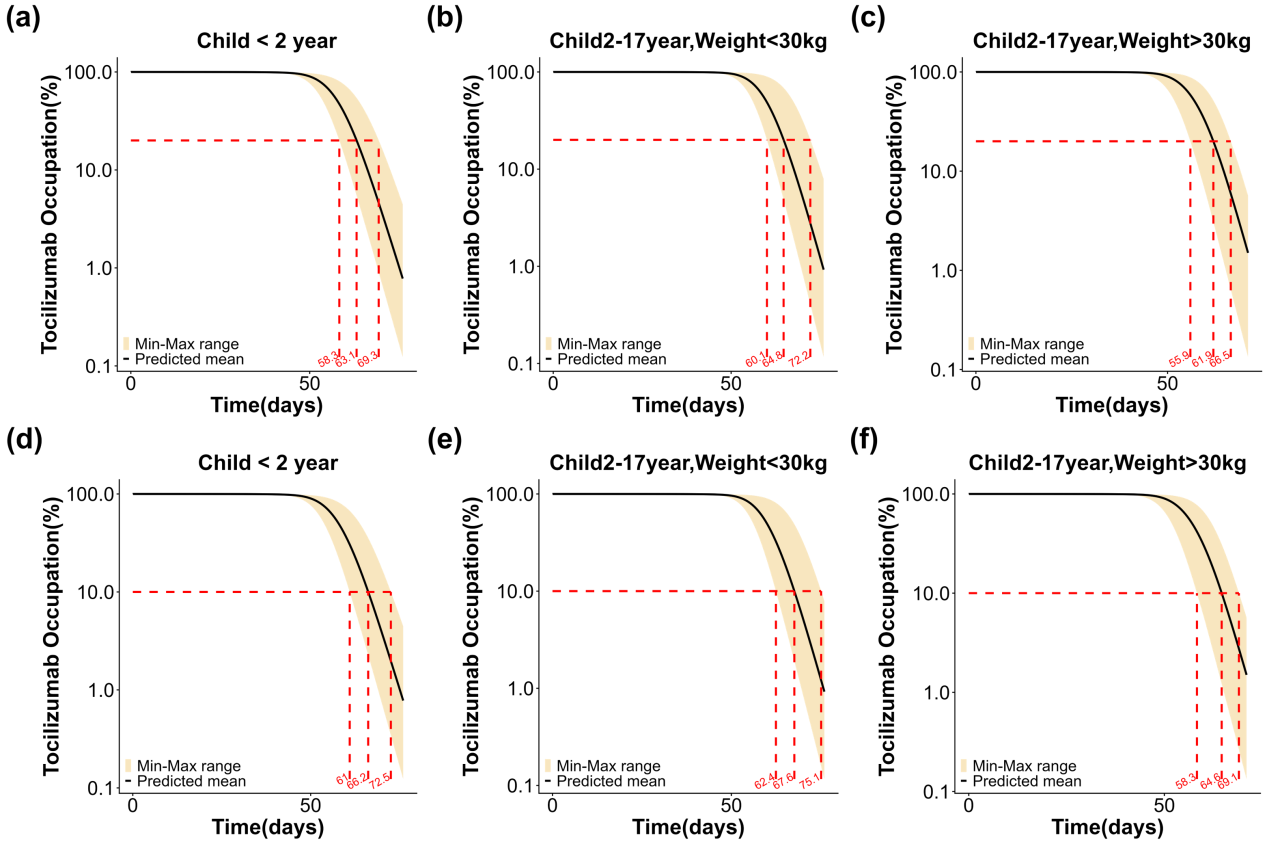


**Supplementary Figure 3**. **Simulated pharmacokinetic and receptor-occupancy profiles for model-informed assessment of tocilizumab washout and dose-escalation strategies in pediatric sJIA patients**. Panels (a–c) show the model-predicted profiles using a TCZ-mediated IL-6R receptor occupancy threshold of 20%, whereas panels (d–f) show the corresponding profiles using a more conservative receptor occupancy threshold of 10%. Panels (a, d) correspond to patients aged <2 years receiving the 6-8-12 mg/kg regimen; panels (b, e) correspond to patients aged 2–17 years weighing <30 kg receiving the 6-8-12 mg/kg regimen; and panels (c, f) correspond to patients aged 2–17 years weighing ≥30 kg receiving the 4-6-8 mg/kg regimen. The solid grey line indicates the predicted mean PBPK profile, and the yellow shaded area represents the minimum–maximum model prediction interval. The horizontal dashed line denotes the receptor occupancy threshold used in each panel.

## Supplementary Figure 4


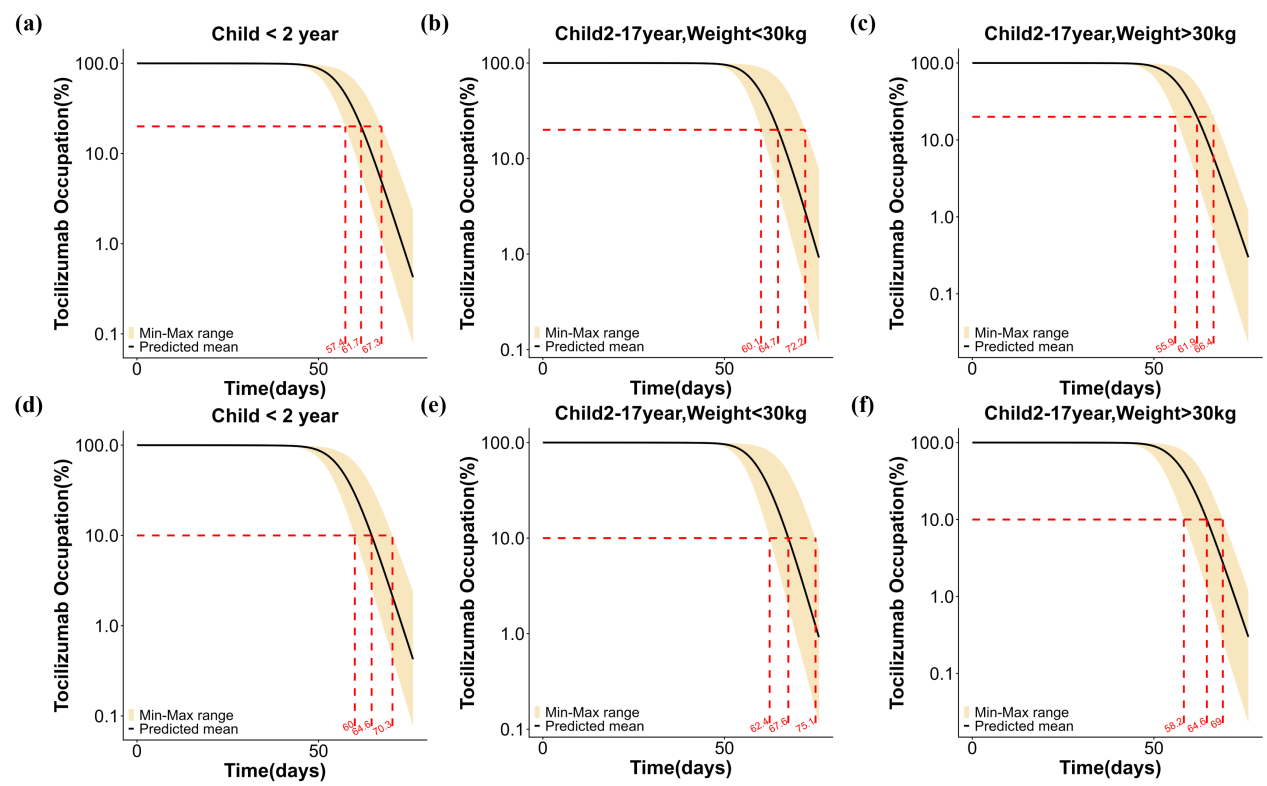


**Supplementary Figure 4. Simulated pharmacokinetic and receptor-occupancy profiles for model-informed assessment of tocilizumab washout following cessation of conventional standard dosing in pediatric sJIA patients.** Panels (a–c) show the model-predicted profiles using a TCZ-mediated IL-6R receptor occupancy threshold of 20%, whereas panels (d–f) show the corresponding profiles using a more conservative receptor occupancy threshold of 10%. Panels (a, d) correspond to patients aged <2 years receiving the conventional 12 mg/kg regimen; panels (b, e) correspond to patients aged 2–17 years weighing <30 kg receiving the conventional 12 mg/kg regimen; and panels (c, f) correspond to patients aged 2–17 years weighing ≥30 kg receiving the conventional 8 mg/kg regimen. The solid grey line indicates the predicted mean PBPK profile, and the yellow shaded area represents the minimum–maximum model prediction interval.

**REFERENCES**

1. Baran P, Hansen S, Waetzig GH, Akbarzadeh M, Lamertz L, Huber HJ, et al. The balance of interleukin (IL)-6, IL-6·soluble IL-6 receptor (sIL-6R), and IL-6·sIL-6R·sgp130 complexes allows simultaneous classic and trans-signaling. *J Biol Chem* (2018) **293**: 6762-75. doi:10.1074/jbc.RA117.001163

2. Willmann S, Höhn K, Edginton A, Sevestre M, Solodenko J, Weiss W, et al. Development of a physiology-based whole-body population model for assessing the influence of individual variability on the pharmacokinetics of drugs. *J Pharmacokinet Pharmacodyn* (2007) **34**: 401-31. doi:10.1007/s10928-007-9053-5

3. Aukland K, Reed RK. Interstitial-lymphatic mechanisms in the control of extracellular fluid volume. *Physiol Rev* (1993) **73**: 1-78. doi:10.1152/physrev.1993.73.1.1

4. Rippe B, Haraldsson B. Fluid and protein fluxes across small and large pores in the microvasculature. Application of two-pore equations. *Acta Physiol Scand* (1987) **131**: 411-28. doi:10.1111/j.1748-1716.1987.tb08257.x

5. Varghese JN, Moritz RL, Lou M, Van Donkelaar A, Ji H, Ivancic N, et al. Structure of the extracellular domains of the human interleukin-6 receptor alpha -chain. *Proc Natl Acad Sci U S A* (2002) **99**: 15959-64. doi:10.1073/pnas.232432399

6. Wang M, Chen L, He J, Xia W, Ye Z, She J. Structural insights into IL-6 signaling inhibition by therapeutic antibodies. *Cell Rep* (2024) **43**: 113819. doi:https://doi.org/10.1016/j.celrep.2024.113819

7. Baran P, Nitz R, Grötzinger J, Scheller J, Garbers C. Minimal interleukin 6 (IL-6) receptor stalk composition for IL-6 receptor shedding and IL-6 classic signaling. *J Biol Chem* (2013) **288**: 14756-68. doi:10.1074/jbc.M113.466169

8. Yu K, Kim B, Shin D, Park MK, Hwang JG, Kim M, et al. Pharmacokinetics and safety of candidate tocilizumab biosimilar CT-P47 versus reference tocilizumab: a randomized, double-blind, single-dose phase I study. *Expert Opin Investig Drugs* (2023) **32**: 429-39. doi:10.1080/13543784.2023.2212155

9. Haranaka M, Eto T, Tanaka T, Yazawa R, Burmester G, Keystone E, et al. Pharmacokinetics and Safety of Intravenous Candidate Biosimilar CT-P47 and Reference Tocilizumab: A Randomized, Double-Blind, Phase 1 Study. *J Clin Pharmacol* (2025) **65**: 233-41. doi:10.1002/jcph.6139

10. Tomaszewska-Kiecana M, Ullmann M, Petit-Frere C, Monnet J, Dagres C, Illes A. Pharmacokinetics of a proposed tocilizumab biosimilar (MSB11456) versus US-licensed tocilizumab: results of a randomized, double-blind, single-intravenous dose study in healthy adults. *Expert Rev Clin Immunol* (2023) **19**: 439-46. doi:10.1080/1744666X.2023.2174104

11. Zhang H, Li X, Liu J, Li C, Wu M, Zhu X, et al. A randomized phase-I pharmacokinetic trial comparing the potential biosimilar tocilizumab (QX003S) with the reference product (Actemra(®)) in Chinese healthy subjects. *Ann Med* (2021) **53**: 375-83. doi:10.1080/07853890.2021.1887925

12. Schmitt C, Kuhn B, Zhang X, Kivitz AJ, Grange S. Disease-drug-drug interaction involving tocilizumab and simvastatin in patients with rheumatoid arthritis. *Clin Pharmacol Ther* (2011) **89**: 735-40. doi:10.1038/clpt.2011.35

13. Burmester G, Trefler J, Racewicz A, Jaworski J, Zielińska A, Krogulec M, et al. Efficacy and Safety of Biosimilar CT-P47 Versus Reference Tocilizumab: 1-Year Results of a Randomised, Active-Controlled, Double-Blind, Phase III Study in Patients with Rheumatoid Arthritis. *Clin Drug Investig* (2025) **45**: 551-63. doi:10.1007/s40261-025-01453-8

14. Smolen JS, Trefler J, Racewicz A, Jaworski J, Zielińska A, Krogulec M, et al. Efficacy and safety of CT-P47 versus reference tocilizumab: 32-week results of a randomised, active-controlled, double-blind, phase III study in patients with rheumatoid arthritis, including 8 weeks of switching data from reference tocilizumab to CT-P47. *Rmd Open* (2024) **10**. doi:10.1136/rmdopen-2024-004514

15. Abdallah H, Hsu JC, Lu P, Fettner S, Zhang X, Douglass W, et al. Pharmacokinetic and Pharmacodynamic Analysis of Subcutaneous Tocilizumab in Patients With Rheumatoid Arthritis From 2 Randomized, Controlled Trials: SUMMACTA and BREVACTA. *J Clin Pharmacol* (2017) **57**: 459-68. doi:10.1002/jcph.826

16. Burmester GR, Rubbert-Roth A, Cantagrel A, Hall S, Leszczynski P, Feldman D, et al. A randomised, double-blind, parallel-group study of the safety and efficacy of subcutaneous tocilizumab versus intravenous tocilizumab in combination with traditional disease-modifying antirheumatic drugs in patients with moderate to severe rheumatoid arthritis (SUMMACTA study). *Ann Rheum Dis* (2014) **73**: 69-74. doi:10.1136/annrheumdis-2013-203523

17. Ogata A, Tanaka Y, Ishii T, Kaneko M, Miwa H, Ohsawa S. A randomized, double-blind, parallel-group, phase III study of shortening the dosing interval of subcutaneous tocilizumab monotherapy in patients with rheumatoid arthritis and an inadequate response to subcutaneous tocilizumab every other week: Results of the 12-week double-blind period. *Mod Rheumatol* (2018) **28**: 76-84. doi:10.1080/14397595.2017.1332507

18. Zhang X, Chen Y, Fettner S, Rowell L, Gott T, Grimsey P, et al. Pharmacokinetics and pharmacodynamics of tocilizumab after subcutaneous administration in patients with rheumatoid arthritis. *Int J Clin Pharmacol Ther* (2013) **51**: 620-30. doi:10.5414/CP201904

19. Mallalieu NL, Wimalasundera S, Hsu JC, Douglass W, Wells C, Penades IC, et al. Intravenous dosing of tocilizumab in patients younger than two years of age with systemic juvenile idiopathic arthritis: results from an open-label phase 1 clinical trial. *Pediatr Rheumatol Online J* (2019) **17**: 57. doi:10.1186/s12969-019-0364-z
